# Supplementary material for: Composite Hydrogel Microspheres Encapsulating Hollow Mesoporous Imprinted Nanoparticles for Selective Capture and Separation of 2′-Deoxyadenosine
Source: Molecules. 2022 Nov 2;27(21):7444. doi: 10.3390/molecules27217444 (PMC9659214; doi:10.3390/molecules27217444)
Supplement: Supplementary file 1 [file molecules-27-07444-s001.zip › molecules-1954087-supplementary.pdf]

Supporting information for

Lu Liu [1], Mengdie Zhou [2], Jianming Pan [1,2]

[1] School of Chemistry and Chemical Engineering, Jiangsu University, Zhenjiang, 212013, China

[2] Key Laboratory of Functional Molecular Solids, Ministry of Education, Anhui Normal University, Wuhu 241002, China

1 Characterization

^{1}H nuclear magnetic resonance spectroscopy (^{1}H NMR) was applied to confirm the structure of AcrU, which was collected with an AVANCEII at 400 MHz (Bruker, Switzerland). The Fourier transform infrared (FTIR) spectra of the samples were tested using a Nicolet NEXUS-470 FTIR apparatus (U.S.A.), which was recorded using KBr pellets for solid samples. The identification of the crystalline phase was performed using an X-ray diffractometer system (XRD) D/max2500VB3+/PC at a scanning rate of 0.02 deg s^{-1}. TGA analysis was performed for powder samples (approximately 50 mg) under a nitrogen atmosphere using a diamond TG/DTA instrument (Perkin-Elmer, U.S.A.) with a heating rate of 5.0 °C min^{-1} from 25 °C to 800 °C. The nanosheet structure of J-SNs-MMIPs was characterized using a transmission electron microscope (TEM, JEOL JEM-2100) and scanning electron microscopy (SEM, JEOL 7800F). XPS spectra were carried out using a thermo ESCALAB 250 with monochromator Al Kα (hν=1486.6eV) for the X-ray sources, and the binding energies were calibrated using the C1 peak at 284.9 eV. Magnetic measurements were carried out using a vibrating sample magnetometer (VSM, 7300, Lakeshore) at 25 °C. The concentration of dA was detected using a UV-Vis spectrophotometer (Shimadzu UV-2450, Japan). High-performance liquid chromatography (HPLC) analysis was performed on an Agilent system (Agilent, 1200, Germany) equipped with a UV-vis detector.

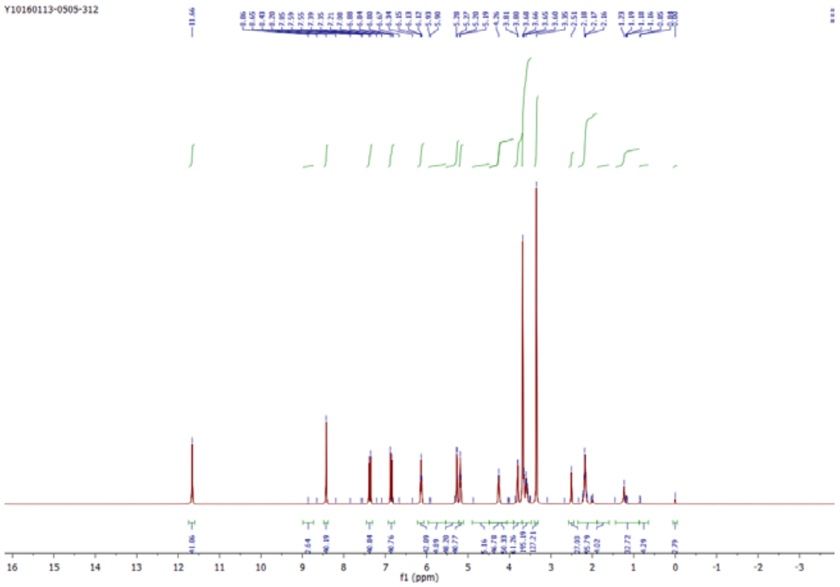

Figure S1. ^{1}H NMR spectrum of AcrU.

2 Study of the binding of functional monomer AcrU to template molecule dA

One bottle of 50 μmol/L dA solution and one bottle of 100 μmol/L AcrU solution were prepared in separate volumetric flasks and set aside. Subsequently, the dA/AcrU mixtures with molar ratios of 50:0, 50:10, 50:20, 50:30, 50:40, 50:40, 50:50, 50:60, 50:70, 50:80, 50:90, and 50:100 were prepared in centrifuge tubes, and the respective absorption peaks were measured using a UV-Vis spectrophotometer after shaking several times to ensure they were fully assembled. This was used to find the optimal binding ratio of dA and AcrU. The result is shown in Fig. S2.

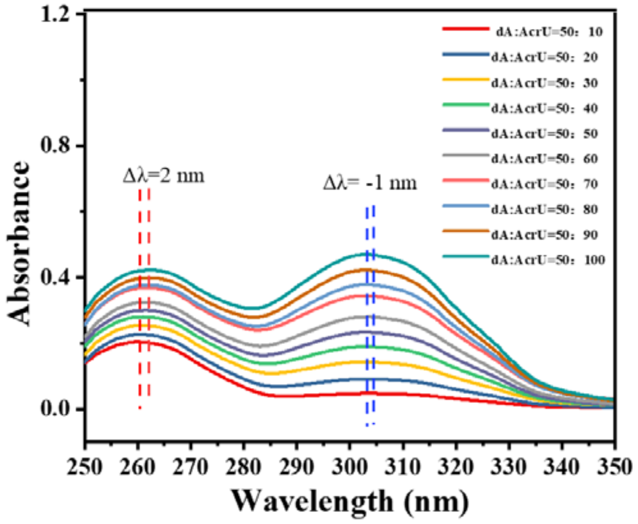

Figure S2 UV-Vis for a series of molar ratios of dA/AcrU mixtures.

3 Batch mode binding experiments

The adsorption performance of MMHSG and NMHSG on dA was evaluated at room temperature. The adsorption experiments were carried out using phosphoric acid buffer solution (PBS) with pH=7.4 as the standard test solution. The amount of adsorbent in a single tube was 5.0 mg and the temperature was 25 °C.

Binding equilibrium experiments: 5.0 mg of adsorbent was added into 5.0 mL PBS solution (pH=7.4) with an initial concentration of dA (C_0=10-1000 μmol L^{-1}). The suspended adsorbent was removed through the membrane after the adsorbent had been in contact with the test solution for 2.0h. UV-Vis was used to detect the concentration of dA in the filtrate. Equation (1) was used to calculate the equilibrium adsorption capacity (Q_e, μmol g^{-1}) at the corresponding concentration.

$$Q_e = \frac{(C_0 - C_e)V}{M} \qquad (1)$$

where C_0 (μmol L^{-1}) and C_e (μmol L^{-1}) are the initial and final concentration of dA in PBS solution, respectively. V (mL) is the volume of dA solution, and W (mg) is the weight of adsorbent.

Binding kinetic experiments: The experimental steps of adsorption kinetics were the same as the above equilibrium experiment steps. The contact time between the adsorbent and the test solution increased successively in the range of 5.0 min–120 min, and the concentration of dA in the test solution was 300 μmol L^{-1}. By using the concentration C_t of the standing time t instead of C_e, the adsorption capacity Q_t (μmol g^{-1}) at time t was also calculated by Equation (1).

Moreover, to measure the selectivity of MMHSG, 5.0 mg of adsorbent was added into the centrifuge tube, and the test solution was PBS (pH=7.4) containing 300 μmol L^{-1} dA, dC, dG, and AMP, respectively. After shaking at 25 °C for 2.0 h, MMHSG was collected by centrifugation, and then the supernatant was filtered through a microporous nitrocellulose membrane (pore size of 0.22 μm). The amount of each compound in the filtrate was then determined by UV-Vis spectroscopy, where the absorption wavelengths of dA, dC, AMP, and ATP were 259 nm, 253 nm, 270 nm, and 260 nm, respectively. The specificity of MMHSG to dA was estimated by imprinting factor (IF) depending on the following Equation (2):

$$IF=\frac{Q_{\mathrm{MMHSG}}}{Q_{\mathrm{NMHSG}}} \tag{2}$$

where Q_{MMHSG} and Q_{NMHSG} (μmol g^{-1}) stand for the adsorption amounts of the template and the competitive compounds onto MMHSG and NMHSG, respectively.
